# Understanding Variation in Adoption of Video Telehealth and Implications for Health Care Systems

**DOI:** 10.18103/mra.v10i5.2751

**Published:** 2022-06-01

**Authors:** Megan E. Gately, Emma D. Quach, Steven D. Shirk, Scott A. Trudeau

**Affiliations:** 1 Geriatric Research Education and Clinical Center, VA Bedford Healthcare System Bedford, MA; 2 Center for Healthcare Organization and Implementation Research, VA Bedford Healthcare System, Bedford, MA; 3 Department of Gerontology, University of Massachusetts Boston; 4 VA Bedford Healthcare System, Bedford, MA; 5 Department of Population and Quantitative Health Sciences, University of Massachusetts Medical School, Worcester, MA; 6 American Occupational Therapy Association, Bethesda, MD

**Keywords:** Telemedicine, health care systems, occupational therapy

## Abstract

**Background:**

Telehealth has rapidly expanded since COVID-19. Veterans Health Administration (VHA), the largest integrated health care system in the United States, was well-positioned to incorporate telehealth across specialties due to existing policies and infrastructure.

**Objectives:**

The objective of this study is to investigate predictors of occupational therapy (OT) practitioners’ adoption of video telehealth.

**Methods:**

This study presents data from a convenience sample of VHA occupational therapy (OT) practitioners administered pre-pandemic, in fall 2019. Survey development was guided by the Promoting Action on Research Implementation in Health Services framework, and gathered clinician attitudes, experiences, and perspectives about video telehealth to deliver OT services. Items included telehealth usage, perceived effectiveness of specific OT interventions, and perceptions about evidence. Our outcome variable denoted practitioners’ level of adoption of video telehealth: telehealth users (adopters), non-users who want to use telehealth (potential adopters and reference group), and non-users who do not want to use telehealth (non-adopters). In multiple multinomial logistic regressions, we tested whether level of adoption was associated with years of VHA work experience and perceived strength of evidence.

**Results:**

Of approximately 1455 eligible practitioners, 305 VHA occupational therapy practitioners participated in the survey (21% response rate). One hundred and twenty-five (41%) reported using video telehealth, whereas 180 (59%) reported not using video telehealth. Among non-users, 107 (59%) indicated willingness to adopt telehealth whereas 73 (41%) were not willing. More VHA work experience predicted higher odds of being an adopter than a potential adopter; perceptions of stronger evidence regarding video telehealth predicted higher odds of being a potential adopter than a non-adopter.

**Conclusion:**

Clinician beliefs and years of experience exerted an influence on clinicians’ use or willingness to use video telehealth. Efforts to enhance adoption of video telehealth should address clinicians’ beliefs regarding the innovative nature of and organizational resources necessary to foster utilization.

## 1 Introduction

Veterans Health Administration (VHA), the largest integrated health care system in the United States ^1^, is a pioneer in telehealth, including video telehealth. Video telehealth is a synchronous, live appointment in which patient and clinician are in different locations. Most video telehealth at VHA has historically been between medical center locations (e.g., urban hospitals and rural clinics), enabled by a telehealth infrastructure which includes facility telehealth staff and equipment across VHA’s broad network of medical centers and community-based outpatient clinics ^2^. In 2018, prior to the pandemic and ensuing public health emergency, VHA set ambitious benchmarks to expand video telehealth into the homes of veterans to increase access to care through the VA Maintaining Internal Systems and Strengthening Integrated Outside Networks Act of 2018 (MISSION Act) ^3^. The MISSION Act, which aimed to strengthen VA health care by creating more care options, broadened telehealth services, beginning with primary care and expanding to include all clinical services, such as specialty care. Occupational therapy (OT), which enables individuals across the lifespan to participate in valued daily activities ^4^, is a specialty care service at VHA. Most VHA OT practitioners are situated at urban medical centers. Thus, in-home video telehealth has the potential to expand the reach of OT services to address the health and wellness of veterans, a population which is aging and has complex care needs ^5^.

There are several barriers to broadscale implementation of video telehealth in the United States, including gaps in accessible technology ^6^, particularly for underserved groups, and a regulatory environment which comprises a patchwork of state-level guidelines ^7^. Individual and system-level factors also influence implementation of telehealth. At the level of the clinician, negative beliefs, such as perceiving telehealth as a threat to one’s role, can influence implementation ^8^, and a lack of education and training in telehealth may also limit implementation ^9, 10^. Regarding system-level factors, organizational support for telehealth is a critical factor and can be an effective tool of recruitment and retention of high-quality clinical staff ^11^. Such investment could be burdensome for smaller facilities ^12^, underscoring the importance of examples from larger health care systems such as VHA to highlight organizational efficiencies and gaps in implementation.

The objective of this study is to investigate factors influencing integration of video telehealth at the largest integrated health care system in the United States. In so doing, this study aims to offer guidance for implementation with identified factors to optimize integration of video telehealth. This study is informed by the Promoting Action on Research Implementation in Health Services (PARIHS), which recognizes that diverse contextual factors, including clinician perspectives, inform the systematic integration of interventions into practice ^13^. PARIHS concepts explicitly guided development of a survey from which these data are derived.

### 1.1 Promoting Action on Research Implementation in Health Services (PARIHS)

**Figure 1.**
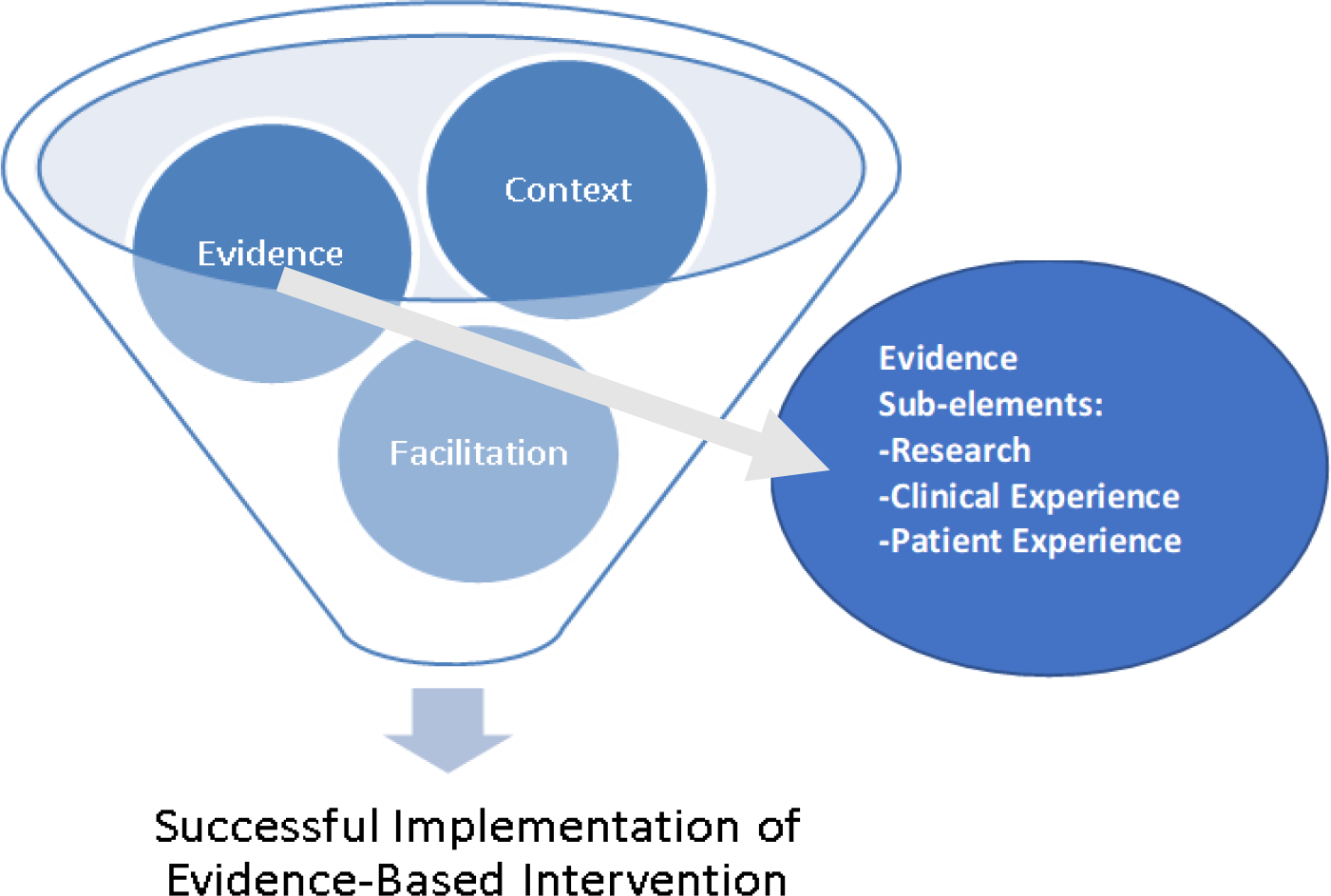
Elements of Promoting Action on Research Implementation in Health Services (PARIHS) framework and Evidence Sub-elements.

The Promoting Action on Research Implementation in Health Services (PARIHS) is a theoretical framework that guides implementation of evidence-based clinical interventions ^14^. PARIHS was developed to support the systematic integration of research findings and other evidence-based practice (EBP) innovations into clinical care to enhance the quality and efficacy of health services ^13^. As a widely utilized framework, PARIHS posits that successful implementation of EBP is a function of three central and interrelated elements: evidence, context, and facilitation. The core element of evidence encompasses multiple sources of information related to the innovation, including published research or guidelines, clinical experience, and patient experience. Socially constructed, evidence includes stakeholder perceptions about the nature and quality of various evidence sources and personal beliefs. Research refers to results of studies utilizing quantitative, qualitative, or mixed methods designs, and published guidelines or recommendations. Clinical experience refers to stakeholders’ experience with the EBP, and respective attitudes, beliefs, history with and motivation toward change. Clinical experience, according to PARIHS, is not necessarily based on available objective evidence but may be tacit or highly subjective. Patient experience includes patient lived experiences, needs, and preferences; it may include exposure to the EBP or related care, personal knowledge, and preferences, which like clinical experience, may not reflect objective research evidence alone. For an overview of PARIHS elements, see Figure 1.

To identify factors influencing implementation of video telehealth at VHA, we utilized PARIHS elements, specifically the evidence element and related sub-elements, research, clinical experience, and patient experience, to develop a survey. Specifically, we utilized the *Successful Implementation Tool* (See Appendix A), a guide which pulls out key PARIHS framework elements into a set of comprehensive tools for implementation trials and evaluation programs using PARIHS. Developed by a PARIHS research team ^15^, the guide is designed to strengthen programs using framework sub-elements by creating a task-oriented guide of how to apply core PARIHS principles ^16, 17^. We used the tool, as well as the Organizational Readiness for Change Assessment (ORCA), an instrument for use by researchers to assist them with operationalizing concepts of the PARIHS framework for implementation studies ^18^, to inform development of survey questions related to clinician perceptions of the evidence base for video telehealth and to investigate the relationship between beliefs and implementation.

## 2 Methods

This study presents data from a national survey of VHA occupational therapy (OT) practitioners about their use of and beliefs towards video telehealth. We examine (1) adoption of video telehealth to deliver OT services, (2) variation in use of video telehealth by clinician demographics, and (3) awareness of evidence (which was broadly conceptualized to reflect PARIHS, per below) regarding use of video telehealth to deliver OT services. Increased knowledge of contextual factors potentially influencing clinicians’ adoption of video telehealth may enhance broadscale integration of video telehealth across health care systems.

### 2.1 Survey Instrument Development and Administration

Development of the 23-item survey was guided by PARIHS elements, particularly clinician attitudes, experience, and perspectives. The survey was developed in a multi-pronged, iterative and collaborative process involving a literature review and input from five VHA subject matter experts with expertise in telehealth, occupational therapy, and geriatrics, including a geriatrics-trained occupational therapist, a behavioral neurologist with extensive experience in geriatrics and telehealth, and VHA’s national Occupational Therapy Discipline Lead. Survey respondents were asked whether they agreed to participate (1 item) and if so, whether they used video telehealth (1 item). If not, respondents were asked whether they would like to use it (1 item). Respondents were then presented a table with a list of thirteen occupational therapy interventions and asked to rate on a four-point Likert scale the perceived effectiveness of (a) in-person service delivery (1 item) of each intervention, and, for those answering yes to using video telehealth, perceived effectiveness of (b) video telehealth delivery (1 item). This question reflected the broad range of occupational therapy interventions delivered at VHA, such as home safety evaluations and supporting participation in instrumental activities of daily living such as medication management. All respondents, regardless of use of video telehealth, rated their comfort level (also on a four-point Likert scale) with video telehealth to deliver each of the thirteen OT interventions (1 item). For a complete list of OT interventions, see Appendix A.

The survey also included ten evidence statements (10 items) adapted from the Organizational Readiness for Change Assessment (ORCA) ^18^, per above. The statements were modified to focus on VHA’s expansion of video telehealth, and asked respondents to rate on a four-point Likert scale agreement with statements related to the evidence for video telehealth. In alignment with PARIHS, statements broadly conceptualized evidence into three main domains: research, clinical experience, and patient experience. Finally, the survey included demographic questions (7 items), including clinician facility, role, years of practice at VHA, years of practice as an occupational therapy practitioner, education, ethnicity, sex, and race. For a complete list of evidence statements and survey items, see Appendix A.

The survey was administered in September and October of 2019 via Research Electronic Data Capture (REDCap), a secure, web-based application designed to support data capture for research studies ^19^. VHA occupational therapy practitioners on VHA’s OT email listservs received an email including the study description and an anonymous URL link to the online survey only available on the VA intranet or via posting to VHA’s online forum for OTs. The link was kept open for four weeks, with three reminder emails and online forum posts sent prior to the survey closing. Emails specified that participation was voluntary, anonymous, and confidential. Complete study details are reported elsewhere ^20^.

### 2.2 Sample Inclusion

This study includes a convenience sample of occupational therapy (OT) practitioners (occupational therapists and occupational therapy assistants) employed at Veterans Health Administration (VHA).

### 2.3 Other Data Source

Rurality geo-coding, developed by the Veterans Health Administration Office of Rural Health to estimate the percentage rurality of the catchment area served by each VA Medical Center (VAMC), was applied to respondents’ primary VAMC.

### 2.4 Outcome Variable

Using data from two survey items, use of video telehealth and desire to use video telehealth, we created three groups delinated by stage of adoption of video telehealth, as informed by the Diffusion of Innovation model ^21^. Group 1 included respondents not currently using video telehealth who indicated not wanting to use it, a group we term “non-adopters.” Group 2 included respondents not currently using video telehealth but who indicated wanting to use it, a group we term “potential adopters.” Group 3 includes respondents currently using video telehealth, a group we termed “adopters.”

### 2.5 Key Independent Variables

VHA tenure, or years of organizational experience, was an ordinal variable in years (0–5, >5–10, >10–20, >20 years). Perceived strength of evidence supporting video telehealth for older adults was measured by summing responses (strongly disagree = 1, disagree = 2, neutral/not sure = 3, agree = 4, strongly agree = 5) to 10 statements on evidence, for a total possible range of 10 to 50. Higher scores represent greater endorsement that video telehealth is evidence-based.

### 2.6 Control Variables

Each model controlled for education, ethnicity, facility’s rurality level, comfort with video telehealth for older adults, and perceived effectiveness of in-person delivery. The level of rurality of respondents’ primary VAMC was a continous variable which was measured in the following categories: 1^st^, 2^nd^, 3^rd^, and 4^th^ quartile, with higher quartiles indicating greater rurality. Ethnicity was specified as a categorical variable (Hispanic, Non-Hispanic, Prefer not to answer as reference group). Practitioners’ highest level of education was an ordinal variable (Associate’s, Bachelor’s, Master’s, or Doctorate degree). Perceived effectiveness of in-person delivery with older Veterans was a categorical variable (*not very effective*, *somewhat effective*, *effective*, *very effective* as the reference group); comfort with using video telehealth with Veterans of all ages was a categorical variable (*not very comfortable*, *somewhat comfortable*, *comfortable*, *very comfortable* as the reference group).

### 2.7 Analysis

We performed descriptive, bivariate, and multivariate analyses. We examined descriptive differences between the groups. We then assessed bivariate correlations among all our variables. Using multinomial logistic regressions, we modeled the outcome (belonging in one of the three adopter groups, with potential adopters, i.e., those who were not using but indicated a desire to use video telehealth, as the reference group) on tenure and perceived strength of evidence and all control variables. We performed 13 multinomial logistic regressions. In each regression, two control variables-- comfort with telehealth and perceived effectiveness of in-person delivery*--*were specific to one of the 13 OT interventions. For example, the regression we term “ADL model” had these variables: tenure, perceived strength of evidence, comfort with ADL intervention delivered over video telehealth, perceived effectiveness of ADL intervention delivered in-person, and other control variables. This analytic approach was designed to provide insights into whether perceptions about specific OT interventions influenced adoption of video telehealth, i.e., whether respondents perceived that certain OT interventions translated better than others to video telehealth (bivariate correlations among the 13 comfort variables showed moderate correlations, as did bivariate correlations among the 13 perceived effectiveness of in-person delivery). All analyses were performed using Stata. Analysis for research purposes was approved by the VA Bedford Health Care System Institutional Review Board (IRB).

## 3 Results

The study sample represents a 20% response rate, with 305 of approximately 1455 eligible VHA occupational therapy practitioners participating in the survey. More than half (180/305, or 59%) reported not using video telehealth and were asked whether they would use it. Of those, under half (73/180, or 41%) indicated not wanting to use video telehealth. The remaining (107/180, or 59%) indicated willingness to adopt telehealth. Table 1 displays sample characteristics by adopter category. Most respondents were female (84%), Master’s-educated (58%) occupational etherapists (92%) with ten years or fewer (54%) of VHA OT practice experience. Respondents were from 107 VAMCs (representing 63% of the 171 VAMCs situated across the United States), which served a Veteran population which was on average 33% rural. There were statistically significant differences amongst adopter categories in gender, ethnicity, and years of practice. Respondent demographics were consistent with American Occupational Therapy Association demographics of OT practitioners in terms of gender, race, ethnicity, and education ^22^.

Table 2 presents multinomial regressions’ odds ratios predicting likelihood of being a non-adopter and adopter (reference group of potential adopters). (Those who were not using video telehealth were asked whether they would or would not use it.) We report results related to evidence beliefs (Table 2 columns denoted potential adopters vs. non-adopters) and then to VHA work experience (Table 2 columns denoted potential adopters vs. adopters).

In predicting non-adopter status, evidence beliefs were consistently a significant variable in all 13 intervention models. Greater perceived strength of evidence was significantly associated with lower likelihood of being a non-adopter compared to potential adopters. For example, in the Leisure intervention model, one additional unit of strength of evidence was associated with 15% lower odds of being a non-adopter. VHA tenure was significant in three models (Home Safety, Rest and Sleep, and Education and Work) but was not significant in 10 of 13 of the remaining intervention models predicting non-adopters. For example, for Education and Work, more years of VHA practice experience was associated with 37% lower odds of being a non-adopter.

When predicting adopters (reference group of potential adopters), in nine of 13 models, VHA tenure was significant in predicting adopter status. (Strength of evidence beliefs was not a significant variable predicting odds of being an adopter.) For example, in the Instrumental Activities of Daily Living (IADL) intervention model, more years of VHA experience were associated with 42% higher odds of being an adopter.

## 4 Discussion

Occupational therapy (OT) practitioners work with clients across the lifespan (womb to tomb) with a variety of challenges that interfere with their ability to successfully participate in day-to-day functional activities. OT practitioners are an integral part of the health care team, providing a comprehensive evaluation that assesses multiple domains, including how physical, cognitive, sensory, emotional, and social functioning affect what clients need and want to do on a daily basis ^23–25^. Comprehensive evaluation results in development of a plan of care which often includes multiple sessions over time to accomplish the clients’ identified goals. The higher the level of impairment, adhering to the recommended plan of care may present a burden to many clients and their families. Thus, some clients may not continue treatment, due to difficulty getting to the medical center. This burden can be mitigated by video telehealth. Given the importance of video telehealth to address access issues, it is important to understand potential barriers and facilitators to intentionally promote expanded utilization.

This study examined findings related to use of video telehealth from a convenience sample of occupational therapy practitioners at the largest integrated health care system in the United States. At the time of the survey (in the fall of 2019), most respondents were not utilizing video telehealth. We were able to gather insights from the majority of those not using video about their potential use of video. By exploring perspectives of VHA OT practitioners at a time when their utilization of video telehealth was generally low, this study offers a lens into the factors driving early adoption and potential adoption of video telehealth removed from the exigent nature of the global COVID pandemic, which limited access to traditional forms of care and thereby forced utilization of telehealth strategies by clinicians who may not otherwise have employed the approach. As such, this study offers a critical window into potential ways to ensure sustained utilization of video telehealth (particularly to serve populations facing access challenges) in tandem with in-person options as the health care context continues to evolve in response to the pandemic.

Regarding predicting adoption status of VHA occupational therapy practitioner survey respondents, evidence beliefs did not predict adoption compared to potential adoption of video telehealth. Thus, OTs’ willingness to use video telehealth before it was mandated by the MISSION Act and before the COVID pandemic was not driven by beliefs about the strength of the evidence. Data from our related study highlighted facilitators to early adoption of video telehealth to be practitioners’ positive beliefs about video telehealth versus organizational factors like supervisor support. This suggests that intrinsic motivational factors may contribute more to the decision to use video telehealth than extrinsic factors like organizational pressures. Individual decisions to adopt a novel intervention appear to be complex and require further investigation.

We also found that more years of VHA practice experience increases the likelihood of OT practitioners adopting telehealth as a service delivery option. More years of practice may imply greater job knowledge, e.g., confidence in skills or increased flexibility in applying skills in new ways, or increased job security. This suggests that the longer one is at an organization, there may be more opportunities for innovative practice. It might also reflect an alignment between the individual employee and the organization’s strategic priorities and values. For example, organizational factors at VHA that encourage innovation include its infrastructure of clinical research, including funded centers of excellence and a stable of clinician scientists ^26^. Thus, the relationship may be both bottom up (driven by clinician factors) and top down (driven by organizational factors). This is a reminder that telehealth programs are influenced by individual-level factors and a broader organizational context ^27^. Further studies should explicate the interplay of these factors using qualitative means.

Regarding the relationship between perceived strength of evidence and adoption of video telehealth, greater belief in the evidence was associated with a lower likelihood of saying ‘no’ when most OT practitioners not using video telehealth were asked, “would you use it?” As a reminder, survey evidence statements, as modified from the ORCA, broadly conceptualized evidence to include results from scientific studies (e.g., randomized control trials), clinical practice experience of self and other clinicians, beliefs about the extent to which video telehealth occupational therapy service delivery aligns with OT practice more generally, and perceived patient beliefs about video telehealth (see Appendix A with evidence belief statements). This finding about the salience of clinicians’ perceptions of the evidence on telehealth increasing their endorsement of it as a strategy aligns with prior research ^28^.

This finding also suggests the potential relevance of clinicians’ actual experience and that of their colleagues in their assessment of an innovation like video telehealth, particularly in the absence of published research studies ^29, 30^. In our related work, we found that while both users and non-users of video telehealth were comfortable using video telehealth for OT interventions, users expressed higher comfort ratings compared to non-users ^20^. This difference speaks to the notion that actual experience with a particular innovation may close the gap between myth versus reality, increasing a practitioner’s likelihood of adopting an innovation ^31^.

This also highlights the need for organizations to continually monitor and assess implementation of video telehealth by gathering the perspectives of clinician users to ensure that it continues to be perceived as beneficial. COVID restrictions on traditional care resulted in a rapid uptake of video telehealth, with clinicians quickly pivoting to integrate telehealth often in the absence of training or established infrastructure. Occupational therapy has seen a rapid growth in telehealth since the pandemic, with OT practitioners using telehealth to provide a range of services, including home health OTs using telehealth to treat homebound clients^32^ and 70% of hand therapists (which include many OT practitioners) reportedly using virtual visits, compared to less than 5% prior to the pandemic^33^. Similarly, a survey by the World Federation of Occupational Therapists during COVID found an increase in telehealth OT service delivery across the world, with reported benefits including positive employee morale and safety^34^.

However, there were challenges to implementation of telehealth which add meaningful context to long-term implementation. Challenges were rife, including difficulty with the technology (e.g., no sound, poor image quality) as well as with the virtual visit workflow, e.g., patients waiting in virtual waiting rooms^35–37^. There were also population-specific barriers for older adults and individuals from rural areas. Barriers to older adult engagement with video telehealth include factors such as lower technical literacy, decreased hearing and sensory processing, and cognitive impairment^38–40^. Regarding rural clients, limitations in resources such as broadband, staffing, and needed technology limit telehealth ^31, 41, 42^, in addition to client cultural factors, including resistance to transitioning traditional forms of treatment to telehealth ^43^. It is not surprising, then, that clients receiving telerehabilitation may be younger and reside in urban settings ^44^. Lastly, while the importance of caregivers to facilitate telerehabilitation is frequently discussed as a facilitator to telehealth, particularly in regards to older adults and those with cognitive impairment ^45–47^, caregiver willingness to participate in telehealth and their support needs are not well-understood ^48^. These are areas in need of further study.

In the current context, the re-opening of health care settings has also prompted a need to identify the ideal mix of telehealth and traditional care going forward; that is, for whom and for what types of care telehealth is suggested. Given the complexity of services which occupational therapy practitioners provide, which include home safety evaluations, training in use of adaptive equipment, provision and evaluation of wheelchairs, group treatment for clients with mental health and other diagnoses, cognitive and low vision rehabilitation, caregiver education and training, and interventions to support sleep, driving, and community mobility ^20, 49–55^, there is a need to develop and disseminate best practices in adapting complex clinical services to telehealth. Challenges unique to OT include the need for adequate visualization of the client and the environment for assessment and to ensure client safety, especially when clinical care requires moving throughout the home, and the need to compensate for the lack of hands-on care. Gaps in available training about how to adapt OT clinical services to telehealth was a primary reported barrier of OT practitioners who utilized telehealth in response to COVID restrictions ^56^. Further research is also needed identify the best match between specific client capacities, the goals of therapeutic intervention, and technology. Further, more outcome studies specific to OT (specifically, comparative and cost effectiveness research) are critical to advance current knowledge ^57^.

To the extent that practitioner beliefs drive implementation of video telehealth, it is necessary that health care settings ensure use of telehealth aligns with individual clinician groups’ standards of practice and core beliefs. Concurrently, there is a need for more published evidence related to OT delivery via telehealth in the areas outlined above to support continued expansion of telehealth opportunities and optimize quality of service delivery. Rapid uptake of telehealth OT in response to COVID has resulted in increased research evidence which may in turn promote further uptake.

## 5 Limitations

There were several limitations to this study. This study included a convenience cohort sample of Veterans Health Administration (VHA) employees, which limits generalizability of findings. While the sample characteristics were similar to occupational therapy practitioners as described by the American Occupational Therapy Association, Veterans Health Administration is a setting which has unique characteristics such as established telehealth infrastructure. As such, findings may not be applicable to non-VA settings. We also did not gather practitioner age, which would have enhanced our understanding of the impact of VHA tenure. Non-respondent bias may limit generalizability, as respondents with strong beliefs may have been more likely to participate in the survey. We also did not include the opportunity to follow up with respondents.

## 6 Conclusion

Overall, this study found that clinicians beliefs and years of practice experience exerted an influence on clinicians’ decision about whether to use video telehealth. Clinicians with stronger beliefs in the evidence and more years of VHA experience were more disposed to opt for video telehealth as a health care delivery option. This aligns with prior work indicating the primacy of clinician attitudes in predicting utilization of health care innovations. Continual assessment of clinician attitudes, and recognition of the importance in workforce training strategies may be indicated to ensure successful, sustained integration of telehealth. More studies are needed to explore the relationship between person-level factors such as age and organizational factors such as availability of technology on implementation of video telehealth.

Video telehealth is clearly here to stay, given that forced utilization of telehealth during the COVID pandemic demonstrated its viability as a strategy to address access challenges for diverse populations. Veterans Health Administration, which was well-positioned to integrate telehealth given its existing infrastructure and policy initiatives, is an ideal setting from which to draw lessons learned regarding sustained integration of telehealth. As health care systems move towards developing best practices in telehealth, the perspectives of stakeholders including front-line clinicians must be understood. Given the importance of clinician attitudes in willingness to use of video telehealth, it is critical that health care systems explicate how both objective and subjective data influence that decision-making process. Understanding how beliefs inform utilization will ensure availability of telehealth as a high-quality option for patient care.

## Supplementary Material

Appendix A. Survey

## Figures and Tables

**Table 1.** Characteristics of three adopter groups

	Non-Adopters ^a^ N=73	Potential Adopters N=107	Adopters N=125	Chi-square	Cramer’s V	P-Value
	% (N)	% (N)	% (N)			
**Gender**				17.88	0.19	< 0.01
Male	10.9 (8)	12.1 (13)	9.6 (12)			
Female	54.8 (40)	73.8 (79)	56.0 (70)			
Non-Binary	0.02 (2)	0	0.01 (1)			
Prefer not to answer	19.2 (14)	0.02 (3)	0.08 (10)			
**Ethnicity**				10.96	0.15	< 0.03
Hispanic or Latino	0.05 (4)	0.07 (7)	0.04 (5)			
Not Hispanic or Latin	54.8 (40)	66.3 (76)	60.0 (75)			
Prefer not to answer	27.3 (20)	11.2 (12)	10.4 (13)			
**Race**				11.73	0.15	< 0.30
American Indian/Alaskan Native	0	0.01 (1)	0.01 (1)			
Asian	0.01 (1)	0.04 (5)	0.04 (5)			
Black/African- American	0.04 (3)	0.07 (8)	0.05 (6)			
Native Hawaiian/Other Pacific Islander	0.02 (2)	0	0			
White	56.1 (41)	61.6 (66)	52 (65)			
Prefer not to answer	23.2 (17)	14.0 (15)	12.8 (16)			
**Role**				0.04	0.01	< 0.98
Occupational Therapist	91.7 (67)	92.5 (99)	92.0 (115)			
Occupational Therapy Assistant	0.08 (6)	0.07 (8)	0.08 (10)			
**Highest Education Level**				8.87	0.06	< 0.35
Associate	0.01 (1)	0.03 (3)	0.03 (4)			
Bachelor	23.2 (17)	28.0 (30)	21.6 (27)			
Master	52.0 (38)	51.4 (55)	40.0 (50)			
Doctorate	0.05 (4)	0.07 (7)	0.08 (10)			
Prefer not to answer	0.05 (4)	0	0.02 (2)			
**Years of VHA practice**				14.51	0.15	< 0.03
Less than 5	39.7 (29)	24.2 (26)	16.0 (20)			
5–10 years	21.9 (16)	31.8 (34)	32.0 (40)			
11–20 years	26.0 (19)	30.8 (33)	36.8 (46)			
≥ 21 years	12.3 (9)	13.0 (14)	15.2 (19)			

a Column percentages may not total 100 due to missing item responses.

**Table 2:** Odds ratios from multinomial regression models examining the association between adoption group membership with perceived evidence and tenure.^b^

	**ADL Model (1)**		**IADL Model (2)**		**Home Safety Model (3)**		**Sensory and/or Cognition Model (4)**		**Veteran/Caregiver Education or Training Model (5)**		**Social Participation Model (6)**		**Leisure Model (7)**	
	**NA**	**A**	**NA**	**A**	**NA**	**A**	**NA**	**A**	**NA**	**A**	**NA**	**A**	**NA**	**A**
Perceived Evidence	0.89 (0.04)**	1.03 (0.03)	0.88 (0.03)**	1.05 (0.03)	0.90 (0.04)*	1.04 (0.03)	0.85 (0.03)**	1.05 (0.03)	0.89 (0.03)**	1.04 (0.03)	0.86 (0.04)**	1.05 (0.03)	0.85 (0.04)**	1.04 (0.03)
Tenure	0.69 (0.15)	1.36 (0.23)†	0.66 (0.15)	1.42 (0.24)*	.58 (0.14)*	1.41 (0.24)*	0.69 (0.15)†	1.41 (0.23)*	.67 (0.15)†	1.45 (0.25)*	.69 (0.16)	1.51 (0.26)*	.68 (0.15)†	1.59 (0.28)**
	**Home Exercise Model (8)**		**Wheelchair Model (9)**		**Durable Medical Equipment Model (10)**		**Rest and Sleep Model (11)**		**Education and Work Model (12)**		**Assistive Technology Model (13)**			
														
Perceived Evidence	0.87 (0.03)**	1.05 (0.03)	0.87 (0.04)**	1.02 (0.03)	0.87 (0.03)**	1.04 (0.03)	0.85 (0.03)**	1.05 (0.03)	0.85 (0.03)**	1.05 (0.03)	0.86 (0.03)**	1.05 (0.03)		
Tenure	0.65 (0.14)†	1.43 (0.24)*	0.68 (0.16)†	1.43 (0.25)*	0.66 (0.14)†	1.37 (0.23)†	0.65 (0.14)*	1.39 (0.24)†	0.63 (0.14)*	1.49 (0.25)*	0.68 (0.15)†	1.38 (0.23)†		

b Cells display odds ratios and, in parentheses, standard errors. Potential adopter group is the reference group for each regression. Perceived evidence ranges between 10–50 denoting level of agreement to 10 statements about evidence about video telehealth. Tenure ranges between 1–4 denoting levels of VA tenure. All 13 models controlled for education, ethnicity, facility’s rurality level, comfort with video telehealth for older adults, and perceived effectiveness of in-person delivery. Statistical significance levels are as follows:

† < 0.10

* P < 0.05

** P < 0.01.

Abbreviation: NA = Non-Adopters. A = Adopters. ADL = Activities of Daily Living. IADL = Instrumental Activities of Daily Living.
