# Supplementary material for: Understanding Variation in Adoption of Video Telehealth and Implications for Health Care Systems
Source: Med Res Arch. Author manuscript; Available in PMC 2022 Nov 17. (PMC9670247; doi:10.18103/mra.v10i5.2751)
Supplement: Appendix A. Survey [file NIHMS1842539-supplement-Appendix_A__Survey.pdf]

1. Do you agree to participate?
2. Do you currently use video telehealth?
3. Would you like to deliver care using video telehealth?
4. Please indicate your comfort (Not comfortable at all; Somewhat comfortable; Comfortable; Very comfortable) with VHA OT practitioners using video telehealth with Veterans to support the following:
    a. ADL (Bathing, dressing, functional mobility, etc.)
    b. IADL (Meal prep, financial management, medication management, etc.)
    c. Home safety
    d. Sensory and/or cognition
    e. Veteran and/or caregiver education or training
    f. Social participation
    g. Leisure
    h. Home Exercise Program/Therapeutic Exercise
    i. Wheelchair clinic/seating and positioning
    j. Durable Medical Equipment provision/follow-up
    k. Rest and sleep
    l. Education and Work (supporting participation in education or work-related activities)
    m. Assistive Technology provision/follow-up
5. Please indicate how effective (Not very effective, Somewhat effective, Effective, Very effective) you find the following OT services when you deliver them in-person.
    a. ADL (Bathing, dressing, functional mobility, etc.)
    b. IADL (Meal prep, financial management, medication management, etc.)
    c. Home safety
    d. Sensory and/or cognition
    e. Veteran and/or caregiver education or training
    f. Social participation
    g. Leisure
    h. Home exercise program/therapeutic exercise
    i. Wheelchair clinic/seating and positioning
    j. Durable Medical Equipment provision/follow-up
    k. Rest and sleep
    l. Education and Work (supporting participation in education or work-related activities)
    m. Assistive Technology provision/follow-up
6. Please indicate how effective (Not very effective, Somewhat effective, Effective, Very effective) you find the following OT services when you deliver them using video telehealth.
    a. ADL (Bathing, dressing, functional mobility, etc.)
    b. IADL (Meal prep, financial management, medication management, etc.)
    c. Home safety
    d. Sensory and/or cognition
    e. Veteran and/or caregiver education or training
    f. Social participation
    g. Leisure
    h. Home exercise program/therapeutic exercise

i. Wheelchair clinic/seating and positioning
j. Durable Medical Equipment provision/follow-up
k. Rest and sleep
l. Education and Work (supporting participation in education or work-related activities)
m. Assistive Technology provision/follow-up

7. For each of the following statements, please rate the strength of your agreement with the statement, from 1 (strongly disagree) to 5 (strongly agree). VHA's expansion of video telehealth:
   a. is supported by randomized control trials (RCTs) or other scientific evidence from the VA
   b. is supported by RCTs or other scientific evidence from other health care systems
   c. should be effective, based on current scientific knowledge
   d. is supported by my clinical experience with VA patients
   e. is supported by my clinical experience with patients in other health care systems
   f. conforms to the opinions of OT colleagues in my setting
   g. has been well-accepted by VA patients in a pilot study
   h. is consistent with OT clinical practices that have been accepted by VA patients
   i. takes into consideration the needs and preferences of VA patients
   j. appears to have more advantages than disadvantages for VA patients
8. In what VA facility do you currently work?
9. What is your role?
10. About how many years you been a VHA OT practitioner?
11. What is your highest level of education?
12. Please select your ethnicity.
13. Please indicate your sex.
14. Please select your race. Select all that apply.
